# Evaluation of anti-obesity activities of ethanolic extract of *Terminalia paniculata* bark on high fat diet-induced obese rats

**DOI:** 10.1186/s12906-015-0598-3

**Published:** 2015-03-24

**Authors:** Ramgopal Mopuri, Muniswamy Ganjayi, Kruthika S Banavathy, Brahma Naidu Parim, Balaji Meriga

**Affiliations:** Department of Biochemistry, Sri Venkateswara University, Tirupati, Andhra Pradesh 517502 India; Department of Molecular Reproduction Development and Genetics, Indian Institute of Science, Bangalore, India

**Keywords:** High fat diet, T*erminalia paniculata*, Lipid profiles, Body composition, Histopathology

## Abstract

**Background:**

The prevalence and severity of obesity and associated co-morbidities are rapidly increasing across the world. Natural products-based drug intervention has been proposed as one of the crucial strategies for management of obesity ailments. This study was designed to investigate the anti-obesity activities of ethanolic extract of *Terminalia paniculata* bark (TPEE) on high fat diet-induced obese rats.

**Methods:**

LC-MS/MS analysis was done for ethanolic extract of *T. paniculata* bark. Male Sprague–Dawley (SD) rats were randomly divided into six groups of six each, normal diet fed (NC), high fat diet-fed (HFD), HFD+ orlistat (standard drug control) administered, and remaining three groups were fed with HFD + TPEE in different doses (100,150 and 200 mg/kg b. wt). For induction of obesity rats were initially fed with HFD for 9 weeks, then, (TPEE) was supplemented along with HFD for 42 days. Changes in body weight, body composition, blood glucose, insulin, tissue and serum lipid profiles, atherogenic index, liver markers, and expression of adipogenesis-related genes such as leptin, adiponectin, FAS, PPARgamma, AMPK-1alpha and SREBP-1c, were studied in experimental rats. Also, histopathological examination of adipose tissue was carried out.

**Results:**

Supplementation of TPEE reduced significantly (P < 0.05) body weight, total fat, fat percentage, atherogenic index, blood glucose, insulin, lipid profiles and liver markers in HFD-fed groups, in a dose-dependent manner. The expression of adipogenesis-related genes such as Leptin, FAS, PPARgamma, and SREBP-1c were down regulated while Adiponectin and AMPK-1alpha were up regulated in TPEE + HFD-fed rats. Furthermore, histopathological examination of adipose tissue revealed the alleviating effect of TPEE which is evident by reduced size of adipocytes.

**Conclusions:**

Together, the biochemical, histological and molecular studies unambiguously demonstrate the potential anti adipogenic and anti obesity activities of TPEE promoting it as a formidable candidate to develop anti obesity drug.

## Background

Excessive energy intake over energy expenditure gives rise to unwarranted growth of adipose tissue leading to obesity [1,2]. Because of its rising prevalence and its association with chronic health disorders such as insulin resistance, hyperlipidemia, hypertension, cardiovascular diseases, non-alcoholic fatty liver and osteoarthritis, obesity has turned out to be a major public health concern in both developed and developing countries [3,4]. Conventional drug therapies of obesity are often associated with adverse side effects and rebound weight gain when drug intake is discontinued. Therefore, research continues to develop safe and effective remedies to treat obesity. In this context, development of natural product-based medication is gaining importance because of its minimum side effects and maximum efficiency.

An array of natural products including plant crude extracts and their bioactive factors have been reported to induce weight loss, prevent diet induced obesity and allied ailments [1]. The possible mechanisms through which such bioactive factors exert their anti-obesity effects include appetite regulation, interfering in lipid digestion and absorption, fatty acid oxidation, regulating lipogenesis-lypolysis and adipogenesis [5]. At molecular level, the major transcription factors such as PPAR γ, C/EBP α, C/EBP β, SREBP-1c and expression of FAS, FABPs, leptin, adiponectin, and AMPK-1α have been implicated in regulating obesity. Previous studies showed that down regulation of lipogenic and up regulation of lipolytic proteins contribute to mitigate obesity and dyslipidemia in high calorie diet-induced rodent model [5]. PPAR family plays essential role in lipid metabolism and is majorly expressed in adipose tissue, liver and skeletal muscle, mediating obesity/anti-obesity signaling events. AMP-activated protein kinase (AMPK) plays a significant role in lipogenesis and fatty acid oxidation through inactivation of acetyl-CoA carboxylase and carnitine palmitolytransferase-1 [3]. SREBPs are important transcriptional factors, involved in regulation of key enzymes of lipogenesis including Acetyl Co-A carboxylase (ACC) and fatty acid synthase (FAS). To make novel therapeutics, targeting lipid metabolism has been considered as a potential and alternative strategy to combat obesity.

*Terminalia paniculata* Roth. (Combretaceae) is a tropical tree with a large natural distribution in western and southern parts of India. Traditionally, its flower juice is used to treat cholera, inflammation of parotid glands and menstrual disorders, cough, bronchitis, cardiac debility, hepatitis, diabetes and obesity [6]. The bark is reported to contain 14 % tannins which have a pyrogallol nucleus along with gallic acid. From the heartwood, phytochemicals such as Ellagic acid, Dimethylellagic acid, Pentamethyl flavellagic acid, Trimethyl flavellagic acid and β sitosterol have been isolated [7]. Previously we have reported the mitigating activity of *T. paniculata* against non alcoholic fatty liver [6]. In the present study, in addition of LC-MS/MS analysis of TPEE, we investigated the attenuating effects of TPEE on body weight, total fat, fat free mass, fat%, serum lipid profiles and the levels of leptin, adiponectin, insulin and liver marker enzymes. We also studied the expression of certain key regulatory genes associated with lipid metabolism in HFD-induced obese rat model.

## Methods

### Collection and extraction of plant material

The bark of *Terminalia paniculata* was collected from Seshachalam forests, Andhra Pradesh, India. Its identity was authenticated by a taxonomist, department of Botany, S.V. University, Tirupati (voucher number 136), and a specimen has been preserved at the departmental herbarium. The bark was shade dried, pulverized to a coarse powder and sequentially extracted with hexane, ethyl acetate, ethanol and water on their polarity basis. The respective extracts were evaporated to dryness in a rotary vacuum evaporator (Buchi) and later different fractions were obtained through column chromatography. Based on phytochemical analysis and preliminary studies, ethanolic extract was selected for further studies [8].

### LC-ESI-MS/MS analysis ethanolic extract of TPEE

LC-ESI-MS/MS analysis of ethanolic extract of *T. paniculata* was carried out on 6520 Accurate Q-TOF (Agilent Santa Clara, CA) mass spectrometer coupled to HPLC equipped with a UV–vis detector. The column used was Zobax SB C18 rapid resolution, 4.6 mm × 150 mm, 3.5 μm particle size (Column-SL, Model G1316B). The conditions were; mobile phase consisted of binary mixture of solvent (A) formic acid (0.1%) and 10 mM ammonium phosphate, (B) acetonitrile + 0.1% formic acid; gradient (in solvent B) with linear gradient programme as follows: Starting from 95% solvent A and 5% solvent B increasing to 35% solvent B over 12 min, then increased to 90% over 35 min., back to 5% solvent B over 1 min and finally isocratic for 10 min. The sample wavelength was set at 240-360 nm. A sample injection volume of 10 μl in methanol and a constant flow rate of 0.3 ml/min were used for the analysis of sample. The mass spectra were acquired with ESI source (Agilent Santa Clara, CA). Nitrogen was used as the sheath and auxiliary gas and helium was used as the collision gas. The ESI MS spectra were acquired in positive ion mode and a spray voltage of 4 kV was employed. The temperature of the heated transfer capillary was 325°C. The mass spectrometer was scanned from m/z 100 to 1200 in full scan mode, gas flow 10 L/min; Nebulizer 40 psi.

### Animals and diets

Male Sprague–Dawley rats (6–8 weeks old), normal pellet diet and high fat diet were obtained from National Centre for Laboratory Animal Sciences (NCLAS), National Institute of Nutrition (NIN), Hyderabad, India. The rats were housed under 22 ± 2°C temperature, 40–60% humidity and 12–12 ± 1 h light–dark cycle and allowed food and water *ad libitum*. High fat diet composition; Beef thallow 29.5 g, Casein 22.0 g, Starch 23.0 g, Cellulose 17.9 g, L- Cystiene 4.0 g, Choline chloride 0.3 g, Vitamin mixture 1.8 g, Mineral mixture 1.5 g were used for this study was obtained from NCLAS, National Institute of Nutrition (NIN), Hyderabad, India.

### Experimental design

Rats weighing 150–170 g were randomly divided in to six groups of six rats each (n = 6). Normal control group of rats received a standard pellet containing required minerals and vitamins, as per AIN-93 guidelines. The other group of rats received HFD during the course of the experimental period (15 weeks). Rats were fed with food and water *ad libetum*. To test the therapeutic activity of *T. paniculata*, TPEE (100, 150 and 200 mg/kg b. wt) was suspended in 0.5% carboxymethyl cellulose and orally administered to the below mentioned HFD-fed rats for 42 days. All procedures involving laboratory animals were followed in accordance with the Institute Animal Ethics Committee regulations approved by the committee (Resolution No: 36/2012–2013/ (i)/a/CPCSEA/IAEC/SVU/MB-MRG).

- Group 1: Normal control group (NC)
- Group 2: High fat diet group (HFD)
- Group 3: HFD + Orlistat 5 mg/kg b. wt
- Group 4: HFD + TPEE 100 mg/kg b. wt
- Group 5: HFD + TPEE 150 mg/kg b. wt
- Group 6: HFD + TPEE 200 mg/kg b. wt

### Determination of food intake, body weight and body composition

Throughout the experimental period the weight gain of rats was monitored weekly and the food intake was monitored daily. At the end of the experiment, blood was collected from overnight fasted animals under inhalation of 2% anesthesia by retro-orbital puncture method. Blood was collected in two different vials, to get plasma or serum by centrifugation at 2500 rpm for 15 min [9].

For the assessment of body composition, anesthetized experimental animals were inserted into total body electrical conductivity (TOBEC) using small animal body composition analysis system (EM-SCAN, Model SA-3000 Multi detector, Springfield, USA). Lean body mass, fat-free mass, total body fat and fat percentages were calculated as described previously [10].

### Oral glucose tolerance test (OGTT) and Estimation of fasting blood glucose and insulin

OGTT was performed at the end of the experiment (end of 15 weeks), after overnight fasting, glucose was administered orogastrically at a dose of 2.0 g/kg b. wt and blood samples were collected from supra orbital sinus at 0,30,60,90 and 120 min. Glucose levels were estimated at all intervals [10].

For estimating fasting blood glucose and insulin levels, rats were fasted overnight and blood was drawn by retro orbital puncture method. Blood glucose was estimated using Kit (Cat No. 1060–500, Stanbio laboratory, USA). Plasma level of insulin was determined using kits obtained from Bio-Merieux, RCS, Lyon, France. Insulin resistance was calculated by homeostasis model assessment.

### Serum lipid profiles

For estimation of lipid profiles, blood samples were centrifuged at 2500 rpm/min for 15 min to separate serum which was then stored at-80°C for further biochemical analysis. Total cholesterol, HDL and triacylglyceride levels were estimated by CHOD-PAP method and GPO-PAP method [11]. LDL levels were calculated by the method of Johnson et al. [12]. The atherogenic index (AI) was calculated by using the method of Muruganandan et al. [13] and Suanarunsawat et al. [14].

$$ \mathrm{Atherogenic}\ \mathrm{Index}\left(\mathrm{AI}\right)=\frac{\mathrm{TC}\hbox{-} \mathrm{H}\mathrm{D}\mathrm{L}}{\mathrm{HDL}} $$

$$ \%\ \mathrm{of}\ \mathrm{Protection}=\frac{\mathrm{AI}\ \mathrm{of}\ \mathrm{H}\mathrm{F}\mathrm{D}\ \mathrm{control}\ \hbox{-}\ \mathrm{AI}\ \mathrm{of}\ \mathrm{treatment}\ \mathrm{group}}{\mathrm{AI}\ \mathrm{of}\ \mathrm{H}\mathrm{F}\mathrm{D}\ \mathrm{control}}\times 100 $$

### Assay of serum AST, ALT and ALP

Aspartate aminotransaminase (AST), Alanine aminotransaminase (ALT), and alkaline phosphatase (ALP) levels were estimated by assay kit methods according to manufacturer’s protocol (Lab Care Diagnostics, India).

### Measurement of leptin and adiponectin

Serum leptin and adiponectin levels were measured by enzyme-linked immunosorbent assay (Crystal Chem, Downer’s Grove, IL, USA), performed in duplicate and were expressed in nanograms per milliliter (ng mL^-1^).

### Fecal lipid extraction and estimation

Fecal matter was collected from the experimental rats at the end of 9th week and 15th week, dried and powdered. Fecal lipids were extracted with chloroform and methanol (2:1), then dissolved in 1% triton × 100 and estimated by standard kit method [15].

### RNA extraction and semi-quantitative PCR

Total RNA was isolated from the adipose tissue of rats by using tri-reagent (Sigma-Aldrich, USA) according to manufacturer’s protocol, and reverse transcribed to obtain cDNA using DNA synthesis kit (A B systems, Foster City, USA). 20 ng of cDNA was used for semi-quantitative PCR with specific primers.

The primer sets used for target genes are Leptin (F: 5’ ATGTGGTACGGAAGGTGGAG3’; R: 5’TGGCTACCTTCGTCTGTGTG3’), AMPK-1α (F: 5’ GGTCCTGGTGGTTTCTGTTG3’; R: 5’ATGATGTCAGATGGTGAATT3’), FAS (F: 5’ GGACATGGTCACAGACGATGAC3’; R: 5’ GTCGAACTTGGACAGATCCTTCA3’), PPARγ (F: 5’TCGGTTTGGGCGAATG3’, R: 5’TTTGGTCAGCGGGAAGG3’), Adiponectin (F: 5’GGTGACCAGGAGATGCT3’, R: 5’ TACGCTGAATGCTGAGTGATA3’), SREBP-1c (F: 5’ GGAGCCATGGATTGCACATT3’, R: 5’ AGGAAGGTTCCAGAGAGGA3’), RPL-19 (F: 5’CGTCCTCCGCTGTGGTAAA3’, R: 5’AGTACCCTTCCTCTTCCCTAT3’).

### Fat pad weights and histological examination

At the end of the experimental period, animals were anesthetized with isoflurane and sacrificed. Adipose tissue (fat pads) from each rat was removed, weighed and stored at-80°C. For histological examination, adipose tissue was fixed in 10% formalin solution and embedded in paraffin. Standard sections of 5 μm thickness were cut, stained using hematoxilin and eosin, viewed under an optical microscope (40X).

### Statistical analysis

Results are expressed as mean ± SD (Standard Deviation). The statistical analysis was carried out by using one-way analysis (ANOVA). The Duncan test was then applied using the program SPSS, Version 18, values with *p* < 0.05 were considered statistically significant.

## Results

### LC-MS/MS analysis

**Figure 1:**
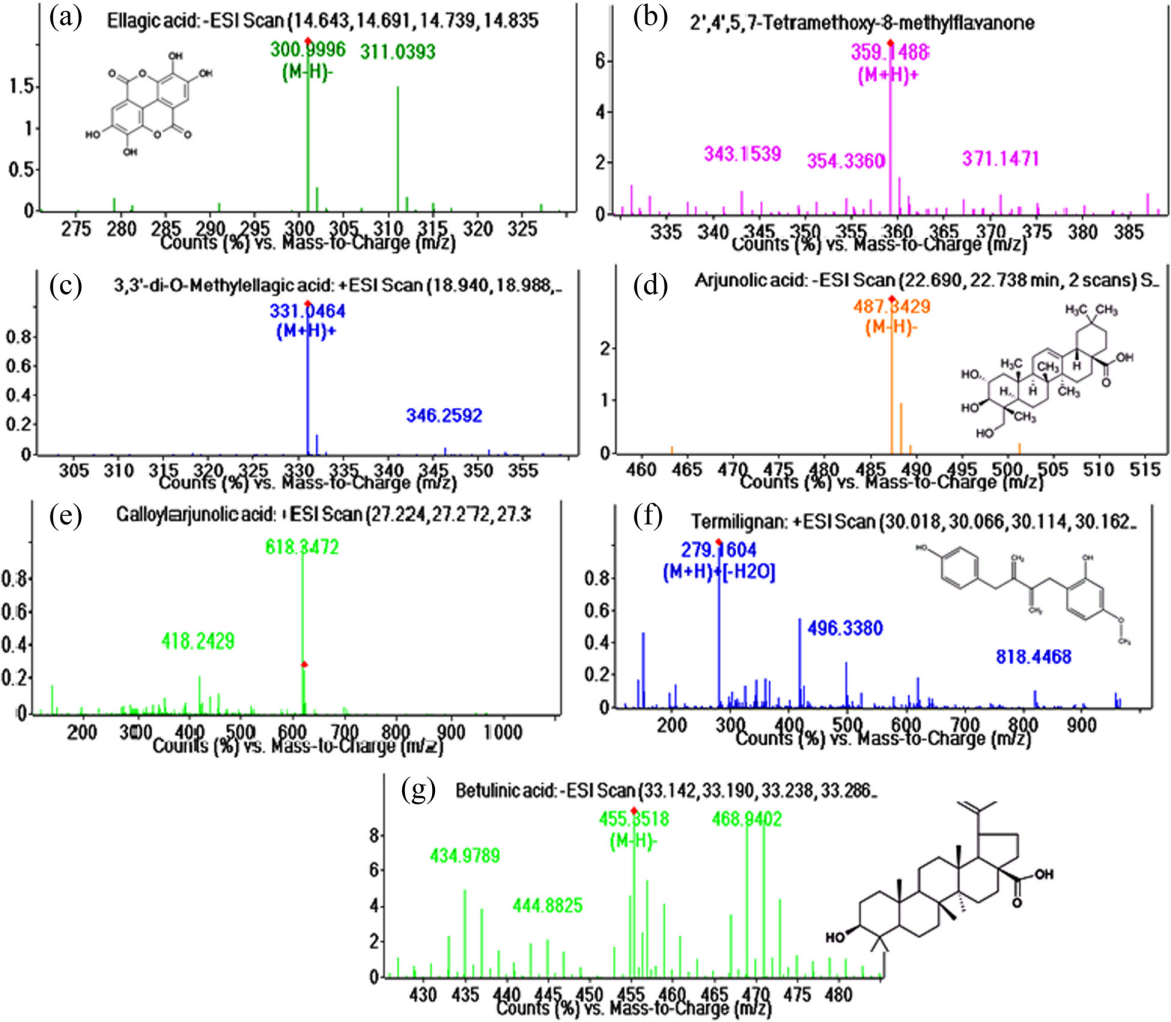
**LC-ESI-MS/MS analysis of ethanolic extract of** ***T. paniculata.*** **a)** Ellagic acid, **b)** 2’,4’,5,7, Tetramethoxy−8-methylflavanone, **c)** 3,3’di-O-Methyl Ellagic acid, **d)** Arjunolic acid, **e)** Galloylarjunolic acid, **f)** Termilignan, **g)** Betulinic acid.

Previously we reported the preliminary phytochemical screening of different solvent extracts of *T. paniculata* [8]. Here we report the LC-Q-TOF-MS/MS analysis of the ethanolic extract of *T. paniculata* and the compounds present in TPEE. The results of the chromatogram are shown in Table 1. The following compounds were identified in TPEE (Ellagic acid, 2’, 4’, 5, 7-Tetramethoxy-8-Methylflavanone, 3, 3’ di-O-Methyl ellagic acid, Arjunolic acid, Galloylarjunolic acid, Termilignan and Butalinic acid). MS/MS Spectra of the mass to charge ratio of ESI scanned spectra results are shown in Figure 1.

**Table 1:** **LC-ESI-MS/MS analysis of ethanolic extract of** ***T. paniculata*** **bark**

**S.No**	**Compound name**	**Retention time**	**Mass**	**Molecular formula**	**MS/MS fragments**
1	Ellagic acid	14.7	302.0068	C14H6O8	300,311
2	2’,4’,5,7, Tetramethoxy				
	−8-methylflavanone	18.08	358.1415	C20H22O6	343,354,359,371
3	3,3’di-O-Methyl				
	Ellagic acid	19.082	330.0.91	C16H10O8	331,346
4	Arjunolic acid	22.746	488.3502	C30H48O5	487
5	Galloylarjunolic acid	27.373	640.3053	C37H52O9	418, 618
6	Termilignan	30.836	296.1641	C19H20O3	279,496,818
7	Betulinic acid	33.24	456.3591	C30H48O3	434,444,455,468

### Effect of TPEE on food intake, body weight and body composition

Food intake, the changes in body weight and body composition of experimental rats are shown in Table 2. A substantial increase in body weights, fat pad weights and a considerable change in body composition were noticed in HFD-fed rats when compared to normal control group rats. However, oral supplementation of TPEE (200 mg/kg b. wt) significantly (*P* < 0.05) reduced the body weight, lean mass, total fat, fat percentage and fat free mass of HFD rats when compared to HFD control group (Table 2).

**Table 2:** **Effect of TPEE on body weights and composition and biochemical parameters of S.D rats**

**Parameters**	**NC**	**HFD**	**Orlistat**	**TPEE (100)**	**TPEE (150)**	**TPEE (200)**
**Physiological parameters**						
† Food intake (g)	20 ± 0.8	24 ± 0.01	19 ± 0.10^*^	22 ± 0.04	21 ± 0.06^*^	21 ± 1.14^*^
‡ Body weight (g)	322.5 ± 14.2	510.8 ± 14.7^a*^	368 ± 9.1^b*^	481 ± 8.5^NS^	456.8 ± 10^b*^	413.6 ± 12^b*^
Lean Mass (g)	307.5 ± 11.2	405.5 ± 13.2^a*^	328 ± 6.8^b*^	400.7 ± 2.8^NS^	393.4 ± 10^NS^	371.2 ± 16^b*^
Total Fat (g)	15 ± 3.5	105.3 ± 12.8^a*^	39.6 ± 5.3^b*^	80.2 ± 8.9 ^NS^	63.3 ± 4.1^b*^	42.4 ± 5.9^b*^
Fat Free Mass (g)	139.2 ± 4.5	178.45 ± 13.8^a*^	147.5 ± 4^b*^	176.5 ± 9.8^NS^	173.6 ± 8.9^b*^	164.7 ± 5.2^b*^
Fat (%)	4.6 ± 0.9	20.56 ± 1.3^a*^	10.7 ± 2.3^b*^	16.6 ± 4.5 ^NS^	13.8 ± 1.2^b*^	10.2 ± 1.8^b*^
**Anti- hyperglycemic index**						
Glucose (mg/dl)	70.9 ± 3.4	153.2 ± 8.9^a*^	96.9 ± 6.2^b*^	130.9 ± 7.3^NS^	125 ± 6.5^b*^	102.4 ± 4.4^b*^
Insulin	2.2 ± 0.2	7.6 ± 0.8^a*^	5.2 ± 0.3^b*^	6.6 ± 0.4^NS^	6.2 ± 0.3^b*^	5.9 ± 0.2^b*^
Insulin resistance	3.3 ± 0.02	5.6 ± 0.01	4.4 ± 0.02	5.2 ± 0.04	4.9 ± 0.05	4.5 ± 0.04
**Organ weights**						
Liver (g)	9.5 ± 0.5	14.7 ± 0.8^a*^	11.7 ± 0.3^b*^	13.5 ± 0.5^NS^	12.3 ± 0.6 ^b*^	11.1 ± 0.3^b*^
Retro peritoneal (g)	13.9 ± 0.8	4.6 ± 1.1^b*^	9.5 ± 0.7^a*^	7.8 ± 0.5^NS^	7.3 ± 0.6^NS^	5.2 ± 0.4^b*^
Mesenteric WAT (g)	1.8 ± 0.4	4.5 ± 0.3^a*^	2.1 ± 0.2^b*^	3.7 ± 0.5^NS^	3.1 ± 0.2^b*^	2.3 ± 0.2^b*^
Epididymal WAT (g)	4.1 ± 0.3	8.4 ± 0.5^a*^	4.4 ± 0.1^b*^	7.8 ± 0.6^NS^	7.0 ± 03^NS^	5.0 ± 0.1^b*^
**Fecal weights and fecal lipids**						
Initial weight	1.0 ± 0.01	1.3 ± 0.102	1.3 ± 0.42	1.3 ± 0.26	1.3 ± 0.035	1.3 ± 1.20
Final weight	1.3 ± 0.24	1.9 ± 0.031^a*^	0.9 ± 0.18^b*^	1.2 ± 0.32^NS^	1.1 ± 0.02^b*^	0.98 ± 0.9^b*^
Fecal lipids (mg/g)						
Initial level	7 ± 2.25	12 ± 1.23	12.5 ± 1.2	11.2 ± 1.6	13.2 ± 0.41	13.0 ± 1.02
Final level	9.8 ± 0.19	12.3 ± 1.2^a*^	16.1 ± 0.2^b*^	12.8 ± 2.5^NS^	14.2 ± 0.98^b*^	15.4 ± 1.08^b*^

The data are given as mean ± S.D (*n* = 6). Values are statistically significant at ^*^ *p* < 0.05 ^a^Significantly different from control, ^b^Significantly different from HFD control, NC: Normal control group, HFD: High-fat diet group, TPEE: *T. paniculata* ethanolic extracts.

The data is analyzed by parametric method-ANOVA.

†: Weighed daily; ‡: Weighed weekly.

### Oral glucose tolerance test

**Figure 2:**
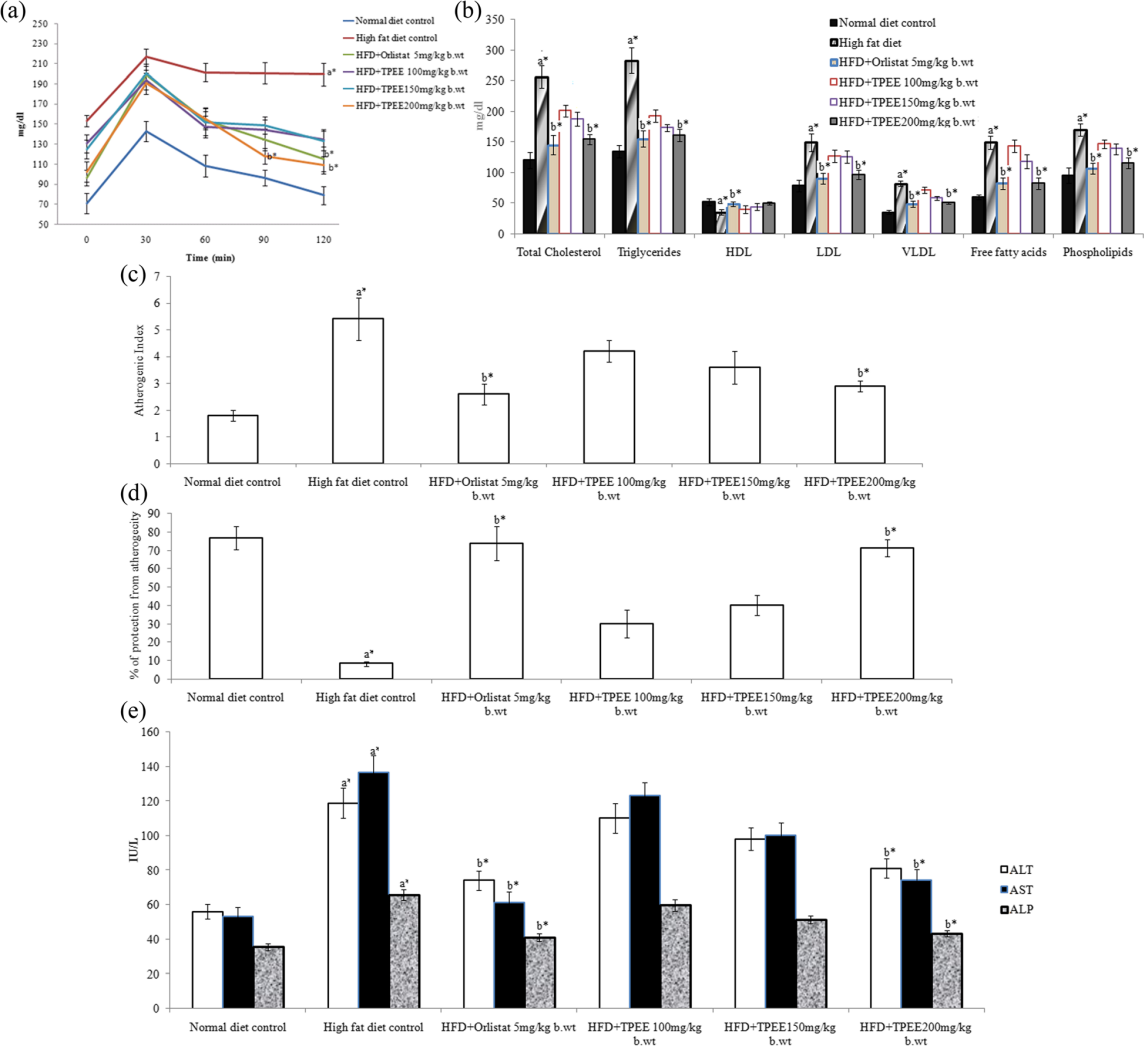
**Effect of TPEE on biochemical parameters of experimental rats. (a)** OGTT **(b)** Serum lipid profiles **(c)** Atherogenic index **(d)** Protection from atherogenicity **(e)** Liver marker enzymes (ALT, AST and ALP) in experimental rats. The data are given as mean ± S.D (*n* = 6). Values are statistically significant at ^*^ *p* < 0.05 ^a^Significantly different from normal control, ^b^Significantly different from HFD control, NC: Normal control group, HFD: High-fat diet group, TPEE: *T. paniculata* ethanolic extracts.

The results of oral glucose tolerance test of the control and experimental obese rats are shown in Figure 2. In normal control rats, maximum elevation in blood glucose level was observed at 60 min after glucose load and declined to near basal level at 120 min, whereas, in HFD-induced obese rats, the peak increase in blood glucose level was noticed even after 60 min and remained high over the next 60 min. Interestingly, supplementation of TPEE or orlistat to obese rats elicited a significant decrease in blood glucose level at 60 min and beyond when compared with HFD control rats (Figure 2a).

### Estimation of Blood glucose, insulin and insulin resistance in TPEE treated groups

Estimation of blood glucose, insulin, insulin resistance, in control and experimental obese rats are depicted in Table 2. There was a significant (*p <* 0.05) elevation in blood glucose, insulin and insulin resistance in HFD induced obese rats over their normal control rats. Oral administration of TPEE tended to reverse the changes in the above parameters in a dose dependant manner, the maximum activity being observed with 200 mg/kg b. wt of TPEE.

### Effect of TPEE on serum lipid profiles

Feeding on HFD resulted in marked elevation of serum total cholesterol (TC), tri glycerides (TG), low density lipoproteins (LDL), very low density lipoproteins (VLDL), phospholipids and free fatty acids, but reduced high density lipoprotein (HDL) levels. However, administration of TPEE (100, 150, 200 mg/kg b.wt) for 42 days to HFD groups has significantly (*P* < 0.05) and dose-dependently normalized the lipid profiles towards healthy atherogenic index (Figure 2b,c,d).

### Effect of TPEE on liver marker enzymes

The activities of liver marker enzymes such as ALT, AST and ALP are depicted in Figure 2e. HFD-fed rats exhibited considerably elevated levels of serum ALT, AST and ALP. However, oral administration of 200 mg/kg b. wt of TPEE has reduced the levels of ALT, AST and ALP by 52%, 51% and 64% respectively.

### Effect of TPEE on leptin and adiponectin

**Figure 3:**
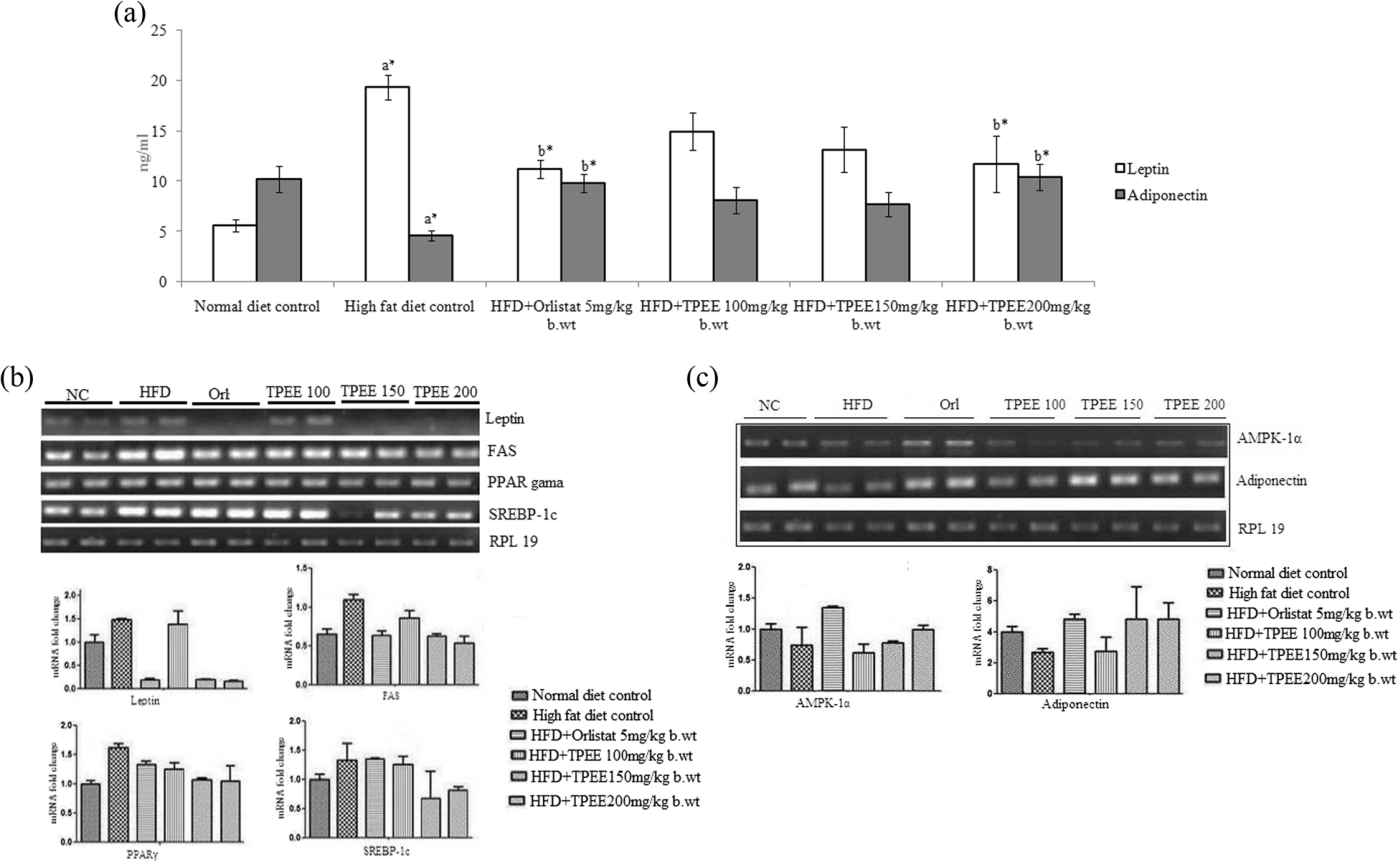
**Effect of TPEE on leptin, adiponectin levels and mRNA expression of obesity associated genes. (a)** Serum leptin and adiponcetin levels **(b)** mRNA expression of Leptin, FAS, PPARγ and SREBP-1c in adipose tissue **(c)** Adiponectin and AMPK-1α expression in adipose tissue of experimental rats.

The levels of serum leptin and adiponectin in control and experimental obese rats are shown in Figure 3a. We noticed a marked elevation in leptin level and decreased adiponectin level in HFD-fed obese rats compared to normal control group. Interestingly oral administration of 200 mg/kg b. wt of TPEE has significantly (*p* < 0.05) decreased the leptin and increased the adiponectin levels in HFD-fed rats.

### Effect of TPEE on fat pad deposition and adipose tissue weights

Vivid differences in fat pad deposition levels were observed in terms of retroperitoneal, mesenteric and epididymal tissues, amongst NC, HFD and TPEE groups. The weights of liver, retroperitoneal, mesenteric and epididymal adipose fat pads were markedly increased in HFD-fed groups. However, administration of TPEE dose-dependently reduced the weight of liver and fat pad weights considerably, the maximum effect being noticed with 200 mg/kg b. wt, as shown in Table 2.

### Effect of TPEE on fecal lipids

Fecal matter was collected from the rectum and wet weight of the feces was mesured. Decreased fecal matter weights were noticed in TPEE adminsitered HFD rats groups when compared to HFD control group. Further. Increased excretion of fecal tirglycerides was observed in orlistat and TPEE treated HFD groups, indicating that TPEE might reduce lipid digestion and absorption (Table 2).

### Effect of TPPE on expression of obesity marker genes

Semi-quantitative PCR analysis of the adipose tissue of HFD-fed rats showed apparently up regulated expression of leptin, FAS, PPARγ, SREBP-1c (Figure 3b) and down regulated expression of adiponectin and AMPK-1α (Figure 3c). On the other hand, treatment with TPEE (100,150 and 200 mg/kg b. wt) had dose–dependently regulated the expression of these genes in HFD-fed obese groups as depicted in Figures 3b and c. More particularly TPEE at a dose of 200 mg/kg b. wt could substantially reverse the expression of respective genes.

### Effect of TPEE on histopathology

**Figure 4:**
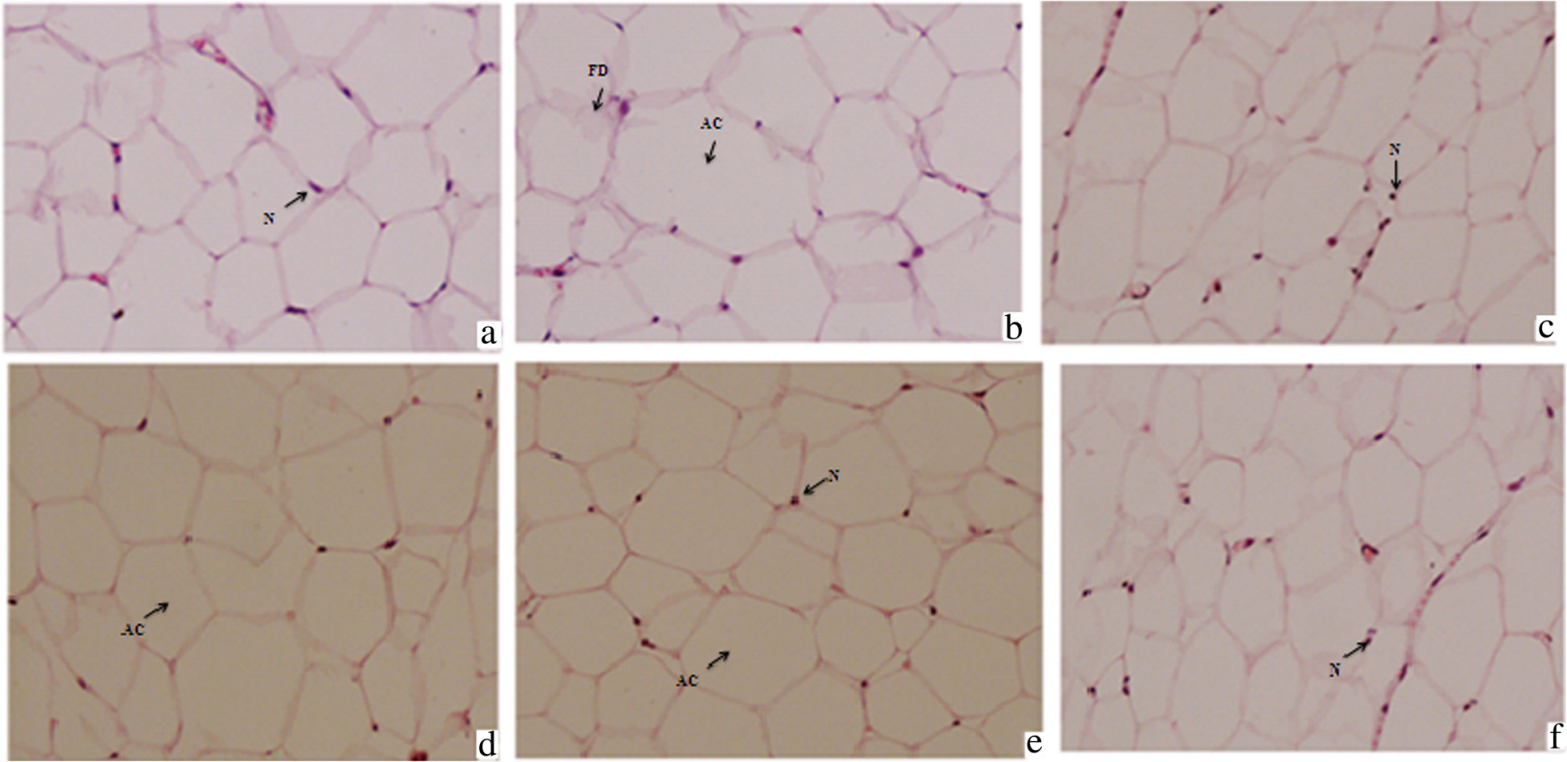
**Histopathology examination of adipose tissue in experimental rats (40X magnifications). (a)** Normal control **(b)** HFD control **(c)** Orlistat 5 mg/kg/ b. wt **(d)** TPEE 100 mg /kg b. wt **(e)** TPEE 150 mg/kg b. wt **(f)** TPEE 200 mg/kg b. wt. N: Nucleus, FD: Fat deposition, AC: Adipose cell.

High fat diet consumption resulted in marked enlargement of adipocytes in HFD control groups. More fat accumulation and consequently marked expansion of adipocytes’ size is shown in Figure 4. Enlarged adipocytes and more fat deposits could be noticed in HFD-fed groups when compared to normal control group. Interestingly supplementation of TPEE (200 mg/kg b. wt) has considerably reduced the size of adipocytes, on par with orlistat.

## Discussion

The prevalence of obesity in recent decades can be attributed largely to changed food habits and increased sedentary life style. In view of the very limited availability of FDA- approved anti-obesity drugs, and considering their side effects, the quest continues to find novel and effective natural product based drugs to combat obesity. Among different targets to treat obesity the ones that can interfere in the process of lipid mobilization (TG levels) and lipid deposition are said to be fundamental [16]. HFD-induced obesity studies in rodents are considered to be a good model as they have been reported to bear close resemblance to human obesity [1]. Rodents fed on HFD developed visceral adiposity, hyperglycemia, dyslipidemia, insulin resistance and hepatic steatosis, which are distinctly linked with human obesity [17]. In the present study we report LC-MS analysis of ethanolic extract of *Terminalia paniculata* (TPEE) and its anti-obesity activities. In our results we showed that dietary fat and lipids have profound effect on the development of obesity in SD rats and this is in concurrence with other studies on high calorie diet induced higher body fat deposition and increased body weight [18,19]. Interestingly oral administration of TPEE has decreased food intake and significantly suppressed higher weight gain, fat percentage, total fat and fat free mass in HFD-fed rats in a dose-dependent manner. This could be possibly owing to TPEE’s role to alter energy expenditure by increasing the expression of lipolytic proteins [20] or inhibition of lipogenesis by reduced expression of FAS and related enzymes [21]. Similar reports of reduced body weight were previously reported with extracts of a few plant species including leaves of *Murraya koenigii*, resin of *Commiphora mukul*, *piper* against HFD-fed obese rats [22,1,23,24].

It is well documented that HFD induced high total cholesterol, glycerides or LDL concentrations are a risk factor for coronary vascular diseases, insulin resistance and non-alcoholic fatty liver [25-27]. Increased free fatty acid or saturated fatty acid incursion from HFD not only produces adipogenesis but also leads to metabolic diseases and chronic activation of inflammation [28]. Such clinical complications of atherosclerosis and obesity could be reduced when serum lipid concentrations are lowered by hypocholesterolemic and anti-obesity agents [29]. In the present study, TPEE administration significantly improved lipid profiles in HFD-fed rats as evidenced by lowered total cholesterol, triglycerides, free fatty acids, LDL concentrations and elevated HDL concentration in serum, towards a healthy atherogenic index. In addition, analysis of fecal matter disclosed elevated fecal lipids in HFD + TPEE treated groups than HFD alone fed group. Increased fecal lipids and decreased serum triacylglycerol levels suggest that TPEE might have anti-lipase activity and also have a role in absorption and transportation of lipids.

Many reports have shown that long term exposure to HFD in experimental animals lead to hyperlipidemia, hyperglycemia and a symptom of insulin resistance [16]. In the present study HFD-induced obese rats developed a hyperglycemic state associated with insulin resistance and/or glucose intolerance. However, supplementation of 200 mg/kg b.wt TPEE showed decreased blood glucose and improved insulin sensitivity in obese rats, as explained by OGTT, possibly by regulating the cell energy metabolism or reducing free fatty acids [30]. The OGTT is advantageous as it is performed under physiological conditions and as it simulates a post absorptive state in which the production and release of insulin and its responsiveness are necessary [24].

Liver function tests are important indicators to reveal the functional status of liver since it is the vital organ involved in detoxification of compounds and in general metabolism. During diet induced obesity, the liver of obese rats displayed characteristic features of hepatic steatosis such as fat accumulation and swelling of rough endoplasmic reticulum and mitochondria in hepatocytes [31]. Increased levels of serum ALT, AST and ALP in HFD group, as shown in the present study indicate alterations in liver metabolic function. TPEE administration has effectively lowered the HFD-induced elevated levels of these hepatic enzymes and lipid profiles demonstrating its protective activity. Our previous studies also showed the therapeutic activity of TPEE against HFD induced fatty liver [6].

Fat cell formation or adipogenesis is a differentiation process by which undifferentiated preadipocytes are converted in to fully differentiated adipocytes which store energy as fat and make the subjects obese [13]. Adipose tissue is a dynamic organ the mass of which changes during lifetime in response to metabolic requirements of the animal, and thus, plays an important role in energy balance. Obesity associated genes such as leptin, FAS, adiponectin, transcriptional factors (PPARs-α, γ, δ, SREBPs, C/EBPs) regulate adipogenesis and lipid metabolism at various stages of adipocyte differentiation [32-34]. It is reported that adiposity is positively correlated to leptin concentration in rodents and humans [35]. Leptin, a protein mainly secreted by the adipocytes, plays a crucial role in regulating body weight by controlling the size of the adipose tissue. It regulates food intake by binding to central nervous system receptors and by modulating the activity of neurons present in appetite control centers of the brain [36]. In our study, down regulation of leptin expression was found in TPEE + HFD groups possibly leading to decreased serum leptin levels which have a direct impact in satiety behavior. The role of leptin may be considered for decreased food intake (though not significant) in TPEE treated groups in our study.

Adiponectin acts as an antagonist of adipogenesis and plays an effective role in regulating lipid and glucose metabolism in insulin sensitive organs in both animals and humans [37]. Low concentration of circulating adiponectin level has been demonstrated in diet induced and genetic models of obesity [38]. It has been reported that up regulation of adiponectin levels is inversely correlated with body fat mass and insulin resistance [39]. Therefore, in our study, up regulation of adiponectin levels in HFD + TPEE administered groups could perhaps ameliorate insulin resistance resulting in reduced blood glucose levels, serum lipid concentrations and body weight loss. PPARγ, an important transcription factor is primarily expressed in adipose tissue and plays an essential role in the regulation of genes involved in adipocyte differentiation, glucose homeostasis and fat tissue development. SREBPs are another family of transcription factors but they are majorly involved in regulation of lipid homeostasis by activating the expression of genes required for the synthesis and uptake of cholesterol, fatty acid and triglycerides. Particularly, SREBP-1c, one of the pro adipogenic transcription factors, induces PPARγ expression and regulates the expression of FAS and AMPK-1α [40-42]. AMPK plays key role in regulation of lipid and carbohydrate metabolism through uptake of glucose. Previous studies have suggested a role for AMPK in the physiological regulation of fatty acid and glucose metabolism, and in the regulation of appetite [34]. In our study, it was found that adipogenic genes such as leptin, SERBP-1c, PPARγ and FAS were up regulated while adiponectin and AMPK-1α were down regulated in HFD-fed groups. However, we have clearly shown that administration of TPEE to HFD-fed groups has considerably reversed the expression of these genes as shown in Figures 3. These results suggest that TPEE could decrease body weight and fat mass by down regulation of SREBP-1c and PPARγ expression, which in turn leads to inhibited expression of lipogenic enzymes including FAS. Further, to confirm the anti adipogenic activity of TPEE, we conducted histopathological examination of adipose tissue. Our results proved the anti-obesity activity of TPEE as evident from the reduced size of adipocytes in TPEE treated groups. Polyphenolic compounds, triterphenoids such as ellagic acid, arjunolic acid and their derivatives present in TPEE might be the major bioactive factors behind its anti-obesity potential. Previous studies demonstrated the anti-diabetic and anti hyperlipidemic activity of elllagic acid, betulinic acid and arjunolic acid from different plant sources [43,44].

## Conclusion

In conclusion, based on physiological, biochemical, histological and molecular analyses we demonstrate that ethanolic extract of *T. paniculata* has potent anti-obesity activities. The anti-obesity effect of TPEE is likely due to reduced expression of lipogenic genes such as FAS, SREBP-1c, PPARγ and leptin and up regulation of adiponectin and AMPK-1α. Finally we suggest that TPEE can be used as a potential therapeutic alternative for the treatment of obesity.

## Abbreviations

HFD: High fat diet
TPEE: Ethanolic extract of *Terminalia paniculata*
TOBEC: Total body electrical conductivity
TC: Total cholesterol
LDL: Low density lipoproteins
HDL: High density lipoproteins
VLDL: Very low density lipoproteins
AI: Atherogenic index
AST: Aspartate aminotransaminase
ALT: Alanine aminotransaminase
ALP: Alkaline phosphatase
FAS: Fatty acid synthase
PPARγ: Peroxisome proliferator-activated receptor γ
SREBP-1c: Sterol regulatory elementary binding protein-1c
AMPK-1α: AMP- activated protein kinase
RPL 19: Ribosomal protein 19
